# Correlation between clinical, ultrasonographic and arthroscopic findings of subscapularis tears: A prospective study

**DOI:** 10.1016/j.jor.2025.04.016

**Published:** 2025-04-28

**Authors:** Arvind Nair, Bishak S. Reddy, Anika Sait, Priya PS, Vivek Pandey

**Affiliations:** a Department of Orthopaedics, Kasturba Medical College, Manipal, Karnataka, India; b Department of Radiodiagnosis, Kasturba Medical College, Manipal, Karnataka, India

**Keywords:** Subscapularis tears, Ultrasonography, Arthroscopy, Clinical tests, Accuracy

## Abstract

**Background:**

Preoperative clinical and ultrasonographic (USG) diagnosis of subscapularis (SSc) tear has always been challenging, with studies reporting varying diagnostic values of these modalities compared with arthroscopy. This prospective study aimed to report the diagnostic values of clinical tests and ultrasonography for SSc tear and to correlate with respect to arthroscopy.

**Methodology:**

Clinical and USG data were collected preoperatively from 144 patients who underwent arthroscopy for rotator cuff tears. Lafosse's classification was used for classifying SSc tear. Sensitivity, specificity, positive predictive value (PPV), negative predictive value (NPV), and accuracy with receiver operating curve (ROC) of clinical tests and USG were statistically analyzed and compared with arthroscopic findings.

**Results:**

Among 144 patients, 70 % had subscapularis tear. Gerber's lift-off was the most sensitive and accurate in diagnosing partial thickness tear (72.1 %, 63.3 %) and overall any tear (77.4 %, 76.7 %). Belly-off was the most specific test in detecting partial-thickness tear (61.5 %) and any tear (88.9 %). Bear hug test (BHT) was the most specific (65.9 %) and accurate (69.8 %) for diagnosing a full-thickness tear. ROC analysis showed maximum accuracy with the belly-off for full-thickness tears and Gerber's lift-off for partial-thickness tears. Compared to clinical tests, USG has lower accuracy in detecting partial tears and higher accuracy (81.7 %) for complete tears.

**Conclusion:**

The diagnostic performance of all modalities for detecting any tear is ranked as follows: Gerber's Lift-Off, Belly Press, Passive Lift-Off, USG, Belly-Off, and BHT. Results of partial tears were similar. For full-thickness tears: USG, Belly-Off, BHT, Passive Lift-Off, Belly press and Gerber's Lift-Off.

**Level of study:**

Level III, Prospective.

## 1 Introduction

One-half of total cuff strength is provided by the largest rotator cuff muscle, the subscapularis.^1^ Furthermore, along with posterosuperior cuff, the subscapularis helps to keep the humeral head centered within the glenoid and significantly contributes to force couple balance.^2^ Tears of the subscapularis compromise this force couple balance.^2^

Surgeons in the pre-arthroscopy era relied on increased passive external rotation and reduced strength of internal rotation to clinically diagnose a subscapularis tear. In 1991, Gerber et al. introduced the Lift-off test, which revolutionized the preoperative diagnosis of the subscapularis tendon.^3^ Further, suboptimal performance of this test in patients with limited passive internal rotation eventually made a pathway for the introduction of the belly press test by Gerber et al., in 1996.^4^ Within a decade, the Internal rotation lag sign by Hertel et al. the belly-off sign by Scheibel et al. and the bear hug test by Barth et al. evolved into practice, which relatively simplified clinical diagnosis of the tear of the subscapularis tendon.^5^, ^6^, ^7^ The belly-off test has been least studied in the literature, while Gerber's lift-off and belly press tests are the most studied ones.^3^^,^^4^^,^^8^, ^9^, ^10^

Based on the trend of diagnostic performance in literature, the bear hug test (BHT) was considered the most sensitive and the lift-off test was deemed the most specific.^5^^,^^8^^,^^10^, ^11^, ^12^, ^13^, ^14^ A recent meta-analysis by Lädermann et al. comprising 13 studies examining diagnostic performance of SSc clinical tests suggested that the BHT was the “most promising test".^13^ Despite these results, variable sensitivity and specificity values were noted for SSc clinical tests in the literature.^5^^,^^8^^,^^10^, ^11^, ^12^, ^13^, ^14^, ^15^

Beyond clinical diagnosis, confirmation with a radiological modality is often required for optimal treatment plan. While MRI has higher sensitivity to diagnose Subscapularis tears,^16^ ultrasonography (USG) with its higher specificity, remains a major diagnostic tool to detect subscapularis tears.^17^ USG has advantage of being cost-effective and real-time visualization of tendons, especially subluxating biceps tendon.^18^^,^^19^ Zhu et al. concluded that USG outperforms MRI in diagnosing partial tears of SSc.^20^

The objective of our prospective study was to assess the diagnostic performances of various clinical tests and USG and compare their performance with arthroscopy.

## 2 Materials and methods

The study was initiated after seeking approval from the Institutional Ethics Committee (IEC/359/2022) and Clinical Trials Registry-India (CTRI/2023/03/051098). 167 patients consecutive patients who underwent arthroscopic rotator cuff repair in our hospital between March 2023 and July 2024 were recruited in this prospective study.

### 2.1 Sample size calculation

A sample size of 144 patients was obtained according to the formula suggested by Lemeshow et al.^21^ assuming a 95 % confidence interval, α error of 5 % with reference to a study by Naimark et al. who reported 30 % incidence of SSc tear in patients with rotator cuff tear.^16^

### 2.2 Inclusion and exclusion criteria

Inclusion criteria were presence of a clinically suspected full thickness rotator cuff tear, which was further confirmed on ultrasonography. Exclusion criteria were secondary frozen shoulder, refusal to consent for preoperative institutional diagnostic ultrasonography, and mini open rotator cuff repair. With exclusion of 23 patients, final study included 144 patients.

### 2.3 Clinical Evaluation

All patients underwent a detailed clinical examination by a single orthopaedic surgeon with subscapularis clinical tests using the classic method described in the literature. The baseline demographic data included the age at presentation (in years), gender, dominant side, and index side. The duration and mechanism of injury with or without a dislocation were also recorded. The five clinical tests for subscapularis used in the study included Gerber's lift-off, passive lift-off, belly press test, belly-off sign, and bear hug test. All the tests were performed with the subject in a standing position.

Gerber's Lift-off test: Before performing Gerber's lift-off test, we confirmed the presence of a full passive internal rotation, wherein patient's hand can easily reach their lower back, to avoid false positive results. The patient was instructed to actively lift the hand off the mid-lumbar spine and advised to hold the hand in this lifted position. If the patient could not complete the test, it was recorded as a positive result.^3^

Passive Lift-off test: To perform the passive lift-off, the subject's elbow was flexed to 90°, the shoulder was extended to 20°, and the arm was passively brought to optimum internal rotation by lifting the dorsum of the hand off the lumbar region by supporting the wrist. When the wrist support was weaned, the subject was advised to hold this position. If unable to maintain the internal rotation, the lag sign was noted, which was recorded as a positive response.^4^

Belly Press test: With the hand pressed flat against the belly, the subject was advised to bring the arm to maximum internal rotation ahead of the coronal plane of the body, and the elbow position with respect to the trunk was observed. The results were recorded as positive if wrist flexion was noted with inability to bring the elbow ahead of the coronal plane of the body or elbow drops back with respect to the trunk.^4^

Belly-off sign: The belly-off sign was performed with the elbow flexed to 90°. With concomitant elbow support, the arm was passively flexed and internally rotated by holding the hand of the subject until the subject's palm rested on the abdomen with the wrist straight, after which the patient was advised to hold the position actively when the wrist support was weaned. If the hand lifted off from the abdomen, it was recorded as positive.^7^

Bear hug test: The bear hug test was carried out with the shoulder brought to 90-degree flexion and elbow in front of the ipsilateral shoulder, maintaining the palm on the opposite shoulder, and the subject was advised to hold in internally rotated position in response to an external rotation force applied perpendicular to forearm.^5^ Difficulty in maintaining an internal rotation or notable weakness was recorded positive response. However, patients with pseudoparalysis were unable to bring the shoulder in 90° flexion. Therefore, to avoid false positive cases, patients with pseudo-paralytic shoulders were excluded to perform Bear hug test.

### 2.4 Ultrasonographic assessment

A single senior musculoskeletal radiologist assessed each patient's rotator cuff using a real-time US scanner (Philips compact ultrasound system series 5500, Netherlands) and a linear ultrahigh-frequency transducer (Philips eL18-4, Ultra-broadband linear array with Pure wave crystal technology), following the protocol outlined in the literature.^18^^,^^19^^,^^22^ Throughout the rotator cuff ultrasonographic assessment, the patient remained in a sitting position. The probe evaluated each rotator cuff tendon in longitudinal and transverse planes. The subscapularis assessment was performed particularly in external rotation, and the findings were recorded as no-tear, partial thickness, and full thickness tear.

### 2.5 Arthroscopic assessment

A single senior shoulder surgeon operated on all patients throughout the study. All patients were operated on in a sloppy lateral decubitus position with the arm suspended using a limb positioner (SPIDER 2 Limb positioner, Smith and Nephew, London, UK). Diagnostic arthroscopy was performed, and all intraarticular pathological findings were noted and dealt with on standard principles. Using the La Fosse classification, the subscapularis tears were graded accordingly. Types 1, 2, and 3 were categorized as partial-thickness tears, and types 4 and 5 as full-thickness tears.^23^ Type 1 were debrided, and all others were repaired with one or more anchors based on standard principles.

### 2.6 Statistical analysis

Data analysis was performed using the SPSS v23 application (IBM Corp. USA). Categorical variables were represented by frequencies and continuous variables by means/standard deviations, medians/IQRs, and percentages. A chi-squared test was employed to measure categorical data. The diagnostic performance of predictors was assessed by computing the following metrics: sensitivity, specificity, positive predictive value (PPV), negative predictive value (NPV), and diagnostic accuracy, using a 2 × 2 cross-tabulation with the results. The degree of diagnostic accuracy (True positive + True negative/n) of a test indicates how reliable is the test to whether the person has disease or not.

The receiver operating curve (ROC) of a test would help distinguish diseased and non-diseased individuals at different cut-off points. The area under the curve was computed, and a test with a value of <0.5 was considered “not useful,” 0.5–0.6 as “bad,” 0.6–0.7 as “sufficient,” 0.7–0.8 as “good,” 0.8–0.9 as “very good” and 0.9–1 as “excellent”.^24^

Using Cohen's kappa score, interrater reliability between various diagnostic modalities was expressed. Value of <0 were considered “disagreement”; 0.10–0.20 as slight agreement; 0.21–0.40 as fair agreement; 0.41–0.6 as moderate agreement; 0.61–0.8 as substantial agreement and 0.81–0.99 as perfect agreement. A threshold of p < 0.05 was maintained for statistical significance.

## 3 Results

In this prospective study on 144 patients who underwent arthroscopic rotator cuff repair, 101 (70 %) had a subscapularis tear on arthroscopic evaluation. The baseline characteristics are summarized in Table 1. 55.5 % (n = 80) of shoulders had pseudo-paralysis, and therefore, were excluded to perform bear hug test (BHT) as BHT cannot be performed accurately in patients with pseudoparalysis.

**Table 1:** Baseline characteristics of the patients. SD, standard deviation.

Variables		Values	
Mean Age (in years) ± SD		55.6 ± 8.22 (range, 41–70)	
Sex		Men: 96 (66.6 %); Women, 48 (33.4 %)	
Dominant side		Right: 132 (91.6 %), Left: 12 (8.4 %)	
Index side		Right: 90 (62.5 %), Left: 54 (37.5 %)	
Etiology	Traumatic: degenerative	84 (58.3 %): 60 (41.7 %)	
Duration of complaint		<2 months	52 (36.1 %)
		2–4 months	26 (18.05 %)
		4 months - 1 year	37 (25.7 %)
		>1 year	29 (20.13 %)
Pseudoparalysis of Index side		80 (55.5 %)	
Clinical tests (positive)		Gerber's Lift off	89 (61.8 %)
		Belly Press Test	85 (59.1 %)
		Passive Lift-off test	81 (56.2 %)
		Belly -off sign	64 (44.4 %)
		Bear Hug test	28 (19.4 %)
Ultrasonographic diagnosis		No Tear	74 (51.5 %)
		Partial Thickness	30 (20.8 %)
		Full Thickness	40 (27.7 %)
Arthroscopic type of Subscapularis tear (as Per Lafosse classification)		No Tear	43 (30.0 %)
		Type 1	46 (31.7 %)
		Type 2	18 (12.5 %)
		Type 3	18 (12.5 %)
		Type 4	12 (8.3 %)
		Type 5	07 (5.0 %)

The diagnostic performances of clinical tests and ultrasonography in comparison with findings of arthroscopy are summarized in Table 2. Gerber lift-off test showed highest sensitivity to detect partial thickness tear (72.1 %) and any tear of the subscapularis (77.4 %). Regarding sensitivity of tests pertaining full thickness tear (FTT), all the tests were highly sensitive (>89 %) to detect a FTT. Maximum specificity for diagnosing a partial thickness tear (PTT) and full thickness tear (FTT) among clinical tests was noted with the belly-off sign (61.5 %) and bear hug test (65.9 %), respectively. Regarding specificity in diagnosing any tear, the belly-off sign showed an optimum specificity value of 88.9 %. The Gerber's lift-off test showed the highest agreement (kappa 0.485) with arthroscopic finding for any tear (Table 2).

**Table 2:** Diagnostic performance of Clinical tests and Ultrasonography and comparison (agreement) with arthroscopic finding.

Modality	Sensitivity	Specificity	PPV	NPV	Accuracy	Kappa measure of Agreement value with arthroscopy	p Value of kappa	Interpretation
**Belly press test (n=120)**								
Partial Thickness tear	**67.6 %**	**51.9 %**	**64.8 %**	**55.1 %**	**60.8 %**	**0.197**	**0.031**	**Slight agreement**
Full thickness tear	**100.0 %**	**46.7 %**	**21.1 %**	**100.0 %**	**53.3 %**	**0.179**	**0.001**	**Slight agreement**
Overall, Any tear	**73.8 %**	**75.0 %**	**87.3 %**	**55.1 %**	**74.2 %**	**0.442**	**<0.001**	**Moderate agreement**
**Bear Hug test (n=53)**								
Partial Thickness tear	**40.0 %**	**52.2 %**	**52.2 %**	**40.0 %**	**45.3 %**	**−0.076**	**0.569**	**No agreement**
Full thickness tear	**88.9 %**	**65.9 %**	**34.8 %**	**96.7 %**	**69.8 %**	**0.339**	**0.003**	**Fair agreement**
Overall, Any tear	**52.5 %**	**84.6 %**	**91.3 %**	**36.7 %**	**60.4 %**	**0.258**	**0.019**	**Fair agreement**
**Belly Off sign (n=120)**								
Partial Thickness tear	**48.5 %**	**61.5 %**	**62.3 %**	**47.8 %**	**54.2 %**	**0.097**	**0.271**	**No agreement**
Full thickness tear	**100.0 %**	**63.8 %**	**28.3 %**	**100.0 %**	**68.3 %**	**0.306**	**<0.001**	**Fair agreement**
Overall, Any tear	**58.3 %**	**88.9 %**	**92.5 %**	**47.8 %**	**67.5 %**	**0.38**	**<0.001**	**Fair agreement**
**Gerber's Lift-Off test (n=120)**								
Partial Thickness tear	**72.1 %**	**51.9 %**	**66.2 %**	**58.7 %**	**63.3 %**	**0.243**	**0.007**	**Fair agreement**
Full thickness tear	**100.0 %**	**43.8 %**	**20.3 %**	**100.0 %**	**50.8 %**	**0.163**	**0.001**	**Slight agreement**
Overall, Any tear	**77.4 %**	**75.0 %**	**87.8 %**	**58.7 %**	**76.7 %**	**0.485**	**<0.001**	**Moderate agreement**
**Passive Lift-Off test (n=120)**								
Partial Thickness tear	**64.7 %**	**53.8 %**	**64.7 %**	**53.8 %**	**60.0 %**	**0.186**	**0.042**	**Slight agreement**
Full thickness tear	**100.0 %**	**49.5 %**	**22.1 %**	**100.0 %**	**55.8 %**	**0.197**	**<0.001**	**Slight agreement**
Overall, Any tear	**71.4 %**	**77.8 %**	**88.2 %**	**53.8 %**	**73.3 %**	**0.437**	**<0.001**	**Moderate agreement**
**Ultrasonography (n=120)**								
Partial Thickness tear	**27.9 %**	**88.5 %**	**76.0 %**	**48.4 %**	**54.2 %**	**0.149**	**0.028**	**Slight agreement**
Full thickness tear	**86.7 %**	**81.0 %**	**39.4 %**	**97.7 %**	**81.7 %**	**0.447**	**<0.001**	**Moderate agreement**
Overall, Any tear	**61.9 %**	**83.3 %**	**89.7 %**	**48.4 %**	**68.3 %**	**0.375**	**<0.001**	**Fair agreement**

**Fig. 1:**
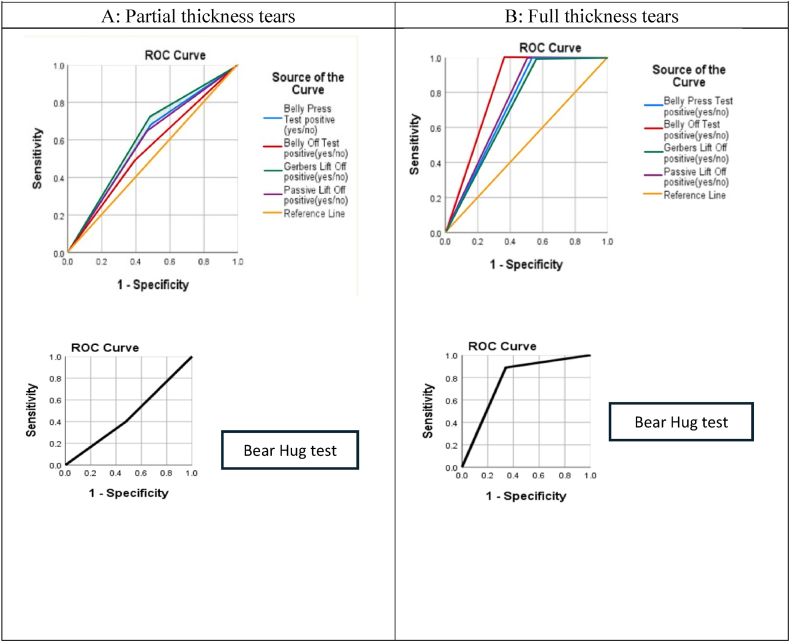
AUC curve analysis for clinical tests. A: Partial thickness tears. B: Full thickness tears.

Ultrasonography (USG) showed a higher sensitivity, specificity, negative predictive value, and accuracy in diagnosing an FTT than PTT. Table 3 mentions the agreement (kappa) between clinical tests and ultrasound. Except for the Passive lift-off test, all other tests showed moderate agreement between clinical tests and USG findings. AUC curve analysis reveals that Gerber's lift-off and Belly-off tests are the most accurate tests to diagnose PTT and FTT, respectively (Table 4, Fig. 1).

**Table 3:** Agreement of clinical tests with ultrasonography to detect different type of subscapularis tears.

USG	Belly press test		Bear Hug test		Belly-Off test		Gerber's Lift -off test		Passive Lift-Off test	
	Kappa score	Agreement	Kappa score	Agreement	Kappa score	Agreement	Kappa score	Agreement	Kappa score	Agreement
**Partial thickness tear**	0.127	**Slight**	0.100	**No**	0.070	**No**	0.105	**No**	0.088	**No**
**Full thickness tear**	0.353	**Fair**	0.396	**Fair**	0.472	**Moderate**	0.321	**Fair**	0.292	**Fair**
**Any tear**	0.52	**Moderate**	0.468	**Moderate**	0.51	**Moderate**	0.47	**Moderate**	0.4	**Fair**

**Table 4:** Receiver operating curve determined area under curve (AUC) for different tests. Values in bold indicate highest AUC among all tests.

Test Result Variable	Partial Thickness Tears		Full thickness tears	
	AUC	p-value	AUC	p-value
Belly Press Test	0.598	0.067	0.733	0.004
Belly Off Sign	0.550	0.346	**0.819**	0.0001
Gerbers Lift Off	**0.620**	0.025	0.719	0.006
Passive Lift Off	0.593	0.082	0.748	0.002
Bear Hug test	0.461	0.628	0.774	0.01

## 4 Discussion

The most significant observations in our study are that the prevalence of SSc tear is 70 % and that Gerber's lift-off test is the “most sensitive and accurate” test to diagnose any tear, whereas the belly-off sign is the “most specific” test to diagnose any tear. Further, Gerber's lift-off and belly-off sign were deemed most accurate in diagnosing PTT and FTT, respectively, as per diagnostic accuracy measured by the ROC curve.

One of the noticeable fact in our study is the higher prevalence of SSc tears (70 %), which is way more than reported in the prevalent literature (29.4 %–46.4 %).^5^^,^^8^^,^^10^^,^^11^^,^^14^^,^^15^^,^^17^^,^^25^^,^^26^ According to Mehta et al. mismatched prevalence results in the literature can be attributed to differing patient populations, age groups, the aetiologies of the tear (traumatic or degenerative), and variable thresholds in modalities for diagnosis of the subscapularis tear.^27^ Another reason for the increased diagnosis of SSc tear is improved arthroscopy techniques and the skills of surgeons who actively look for SSc tears.^17^

Before we discuss the diagnostic performances of various clinical tests, we must discuss the effect of pseudopalsy over the Bear hug test affecting the performance of this test in our series. To perform a classic bear hug test, the forward flexion of the shoulder up to 90° is a must, which is difficult in pseudopalsy. Scheifer et al. observed 11 false positive results of the bear hug test in their study in 49 shoulders, the reason for which, as justified by them, is a concomitant supraspinatus tear that would be activated in forward elevation of the shoulder or a biceps tendinopathy.^28^ Further, Jang et al. considered pseudo-paralysis and stiffness as the primary exclusion criteria while studying the correlation between subscapularis tears and clinical tests.^29^ We encountered 80 (55.5 %) pseudo-paralytic shoulders in our study, which was the sole reason for the reduced performance of the bear hug test. However, there are two views about performing Bear hug test (BHT) in two positions, 45 and 90 degrees of shoulder flexion. In an electromyographic (EMG) study on SSc muscle, Chao et al. concluded that BHT at 45^0^ and 90^0^ shoulder flexion provides valuable information about upper and lower SSc muscle, respectively.^30^ In contrast, Pennock et al. in another EMG study concluded that no typical position of arm would activate any part specific part of SSc while performing BHT.^31^

### 4.1 Sensitivity of various clinical tests

Our study concluded that Gerber's lift-off test is one of the most sensitive (77 %) tests for diagnosing “any SSc tear” (partial or complete) with respect to the gold standard arthroscopic finding.

However, majority of the literature reported that BHT is most sensitive test (45.4 %–74 %) to diagnose any SSc tear.^5^^,^^10^^,^^11^^,^^13^^,^^14^ Nevertheless, Bartsch et al. reported belly press test to be most sensitive (88 %).^8^ Nevertheless, contrary to our finding, the existing literature reports that the Gerber's lift-off is the most specific test.^5^^,^^10^, ^11^, ^12^^,^^15^

### 4.2 Specificity of various clinical tests

Our study confirmed that the belly-off sign was highly specific among all clinical tests in diagnosing any SSc tear, which agreed with the observations of Bartsch et al. (87 %).^8^ However, rest of the literature reported different tests with higher specificifity. While Gerber's Lift-off test was considered the most specific according to Barth et al. (100 %), Yoon et al. (100 %), Kappe et al. (98 %), and Rhee et al. (95.6 %), Takeda et al. reported bear hug test to be more specific (97 %).^5^^,^^10^^,^^11^^,^^14^^,^^15^ Somerville et al. reported optimum specificity with the Belly press test (97 %) and equal specificity values among the Gerber's lift-off (96 %) and passive lift-off (96 %).^25^ Observing the above mentioned values, it appears that almost all subscapularis tests show high specificity values, with insignificant differences between them.^5^^,^^8^^,^^12^^,^^13^

### 4.3 Predictive value (positive and negative) of various tests

In our study, compared with arthroscopy, the PPV of all clinical tests to diagnose a PTT was greater than diagnosing a full-thickness tear whereas NPV was higher for FTT. To diagnose any SSc tear, the highest PPV was noted with the belly-off sign (92.5 %), which was in solidarity with observations of Bartsch et al. ( ).^8^

Furthermore, the most significant observation in our study with respect to NPV is that all clinical tests showed almost 100 % NPV for FTT, implying that the patients who were tested negative by the tests did not have a full-thickness tear.

Most of the prevalent literature has not distinguished and compared the predictive value of clinical tests independently for full-thickness and partial-thickness tear of the subscapularis; hence, our findings turn out to be unique in this context.^5^^,^^8^^,^^10^^,^^12^, ^13^, ^14^, ^15^^,^^25^

Most clinical tests showed a high NPV in almost all existing literature except for Yoon et al.^15^ The literature that exhibits all clinical tests showing excellent NPV was Rhee et al.‘s.^10^

### 4.4 Accuracy of tests with ROC analysis

Upon performing the ROC analysis, we observed the highest area under the curve (AUC) among all tests for Gerber's lift-off to diagnose a partial thickness tear and the belly-off sign to diagnose a full-thickness tear. The value of AUC for Gerber's lift-off was 0.62, indicating a “sufficiently accurate” test for partial thickness tear detection, and 0.819 for the belly-off sign, indicating “very good” diagnostic precision for full thickness tear detection. With this parameter being analyzed, the clinical test for partial thickness tears could be ranked easily as follows: Gerber's lift-off (0.62), Belly press (0.598), Passive lift-off (0.593), Belly-off sign (0.55), and Bear hug test (0.461). For full-thickness tears; Belly-off sign (0.819), Bear hug test (0.774), Passive lift-off (0.748), Belly press test (0.773), and Gerber's lift-off test (0.719).

Our observations closely align with those of Rhee et al. who reported the following ranking for a full-thickness tear: Belly-off sign (0.836), Bear hug test (0.82), Gerber's Lift off test (0.77), Passive lift-off (0.72), and Belly press test (0.65).^10^ Other than Rhee et al. no other author has compared the accuracy of the belly-off sign. However, Yoon et al. observed Gerber's lift-off with an “excellent” diagnostic accuracy and bear hug test as the least.^15^ Regarding the kappa agreement score for assessment of interrater reliability, all clinical tests agreed with arthroscopy for a full-thickness tear. Compared to other clinical tests, the belly-off sign most agreed with ultrasonography.

Ultrasonography proved an efficient radiological modality for detecting a full-thickness tear. The overall diagnostic accuracy of ultrasound in detecting any tear was 68.3 %. The specificity of ultrasound was significantly higher for diagnosing a partial-thickness tear (88.5 %) than a full-thickness tear (81 %). This trend of high specificity was observed in the majority of the literature, including Dekker et al. (97 %), Narasimhan et al. (93.1 %), Singisetti et al. (100 %), and Toprak et al. (100 %).^17^^,^^32^, ^33^, ^34^

The sensitivity and specificity of USG for detecting partial thickness tears were 27.9 % and 88.5 %, respectively. These results are consistent with those reported in studies by Narasimhan et al. and Dirkx et al.^17^^,^^35^ In contrast to partial-thickness tears, USG had a good diagnostic accuracy of 81.7 %, sensitivity of 86.7 %, specificity of 81 %, and specificity of 81 % for diagnosing full-thickness tears. The sensitivity of 93.5 % and the specificity of 75.6 % reported by Sizsheng Zhu et al. were nearly in line with these findings.^20^

### 4.5 Limitations of the study

This prospective study has limitations. Firstly, the clinical examination was performed by a single senior orthopedic surgeon trained to assess the findings, and these were not compared with the findings of another surgeon, which resulted in a lack of interobserver variability. Secondly, the USG findings could not be verified by another radiologist due to a lack of time, funds and consistency regarding the experienced radiologist. Thirdly, since the presence of pseudo-paralysis affects the performance of the Bear Hug test, the pseudo-paralytic patients did not undergo the Bear Hug test, which might have affected our results concerning the performance of the Bear Hug test.

## 5 Conclusion

Gerber's lift-off is the most sensitive test overall and was deemed sufficient to diagnose a partial thickness tear of subscapularis. The belly-off was the most specific test overall and showed high accuracy in diagnosing a full-thickness tear of the subscapularis. Our study suggests that no single clinical test prevails to diagnose all tears equally.

USG had a high sensitivity, specificity, NPV, and diagnostic accuracy to diagnose a full-thickness tear and a high specificity in diagnosing a partial-thickness tear. Based on our observations, the performance of all modalities in terms of diagnostic accuracy to diagnose any tear can be ranked as follows: Gerber's Lift Off, Belly press, Passive Lift Off, USG, Belly Off, and Bear Hug test. For Partial thickness Tears, the ranking is as follows: Gerber's Lift Off, Belly press test, Passive Lift Off, USG similar to Belly Off sign, and Bear Hug test. For Full-thickness tears, the ranking is as follows: USG, Belly Off sign (almost similar to USG), Bear Hug test, Passive Lift Off, and Gerber's Lift Off test.

## CRediT authorship contribution statement

**Arvind Nair:** Data curation, Formal analysis, primary Manuscript preparation. **Bishak S. Reddy:** Data curation, Formal analysis, primary Manuscript preparation. **Anika Sait:** Writing – review & editing, Manuscript revision and editing. **Priya PS:** Ultrasonography, Data curation. **Vivek Pandey:** Conceptualization, Methodology, Supervision, Validation, Writing – review & editing, Review, and editing of the manuscript.

## Patient consent

The patients consent was obtained for the study.

## Financial remuneration to the authors

None.

## Ethical committee

Institutional Ethical Committee, Kasturba Hospital, Manipal (Registration no- ECR/146/Inst/KA/2013/RR-13) approval was obtained with a study number - IEC/359/2022.

## Clinical trial registry

Clinical Trial Registry India- 2023/03/051098.

## Ethical approval

IRB approval and the approval number- IEC approval obtained - IEC/359/2022.

The study has been conducted in accordance with the ethical principles mentioned in the Declaration of Helsinski (2013)

## Funding information

The study is not funded by institution or any other agency. Authors have not received any grant for the same.

## Conflict of interest of any of the authors

None.
